# Editorial: Glial interactions in glaucoma

**DOI:** 10.3389/fopht.2024.1393555

**Published:** 2024-05-06

**Authors:** Youichi Shinozaki

**Affiliations:** Department of Diseases and Infection, Tokyo Metropolitan Institute of Medical Science, Tokyo, Japan

**Keywords:** glia, glaucoma, astrocytes, microglia, Müller cells

This Research Topic gathers different contributions highlighting glial cells in the pathogenesis of glaucoma. In the pathogenesis of glaucoma, the focus of research on the mechanisms underlying the development of the condition has traditionally centered around the molecular mechanisms within retinal ganglion cells (RGCs), primarily affected in glaucoma. However, similar to neurodegenerative disorders of the brain, glial cell changes, termed activation, have been increasingly recognized as common in glaucoma. Some evidence now suggests that abnormalities in glial cells might even serve as triggers for the onset of glaucoma.

This Research Topic aims to enhance understanding of the pathogenesis of glaucoma with a primary focus on glia. Four comprehensive reviews from different research groups are presented.

Elevated intraocular pressure, commonly observed in primary open-angle glaucoma (POAG), is well-known to induce inflammation and lead to damage in RGCs. Ishikawa et al. shed light on microglia, immune cells in the central nervous system, discussing their role in neuroinflammation and glaucoma development. They explore intriguing discussions on microglia-astrocyte interactions causing neuroinflammation and the potential of targeting microglial neurotoxicity for glaucoma treatment.

Cullen and Sun provide an overview focusing on astrocytes in the retina and optic nerve head in the context of glaucoma pathogenesis. Astrocyte alterations, i.e., activation, are known in glaucoma and various neurodegenerative diseases. However, the discussion on the diversity of astrocytes in ocular tissues, such as the retina, optic nerve head, and optic nerve, has been scarce. The authors delve into a fascinating discussion on the functions of ocular astrocytes, particularly regarding their role through gap junctions.

Masson et al. introduce various insights into the role of lipids, particularly cholesterol and oxysterols, in the neuro-glial interactions within the retina. Among glial cells, Müller cells, specialized macroglia in the retina, play a crucial role in maintaining retinal cholesterol homeostasis. The manuscript also explores the potential contribution and mechanisms of Müller cell activation related to abnormal sterol metabolism in neurodamage and glaucoma onset.

Shinozaki et al. discuss the potential contributions of oligodendrocytes in glaucoma onset, expanding the focus beyond microglia, astrocytes, and Müller cells. They engage in an interesting discussion on novel approaches to glaucoma treatment targeting glia, such as gene therapy, pharmacological control, cell transplantation, corneal electrical stimulation, and extracellular vesicles.

Currently, glaucoma treatments are largely limited to approaches that lower intraocular pressure. Glial cells are widely distributed throughout the nervous system, including ocular tissues. As highlighted in this Research Topic, pathological changes in glial cells play an essential role in inducing damage to RGCs, leading to visual impairment. On the other hand, glial cells exhibit diversity, and neuroprotective glia also exist. The proper regulation of glial cell function is considered an attractive therapeutic target for addressing glaucoma, as neuroprotective glia contribute essential neurotrophic factors for neuroprotection and neural function recovery. Leveraging cutting-edge technologies allows for precise control of glial function, effectively suppressing neurotoxicity while enhancing neuroprotection and regeneration. Several glia-related molecules have already advanced to clinical trials, and further progress is anticipated in drug development targeting glial cells. The advancement of drug development targeting glial cells holds great promise.

## Author contributions

YS: Writing – original draft, Writing – review & editing.

